# *Lactobacillus sp.* attenuates oral and hepatic alterations and decreases caspase-8 expression in ligature-induced periodontitis

**DOI:** 10.1590/1678-7765-2026-0143

**Published:** 2026-06-18

**Authors:** Victor Lucas Ribeiro Lopes, Even Herlany Pereira Alves, Alan Leandro Farias de Carvalho, Vinícius da Silva Caetano, Hélio Mateus Silva Nascimento, Maria Vitoria Pereira de Sousa, Paulo Roberto Carneiro Gomes, Any Carolina Cardoso Guimarães Vasconcelos, André Luiz dos Reis Barbosa, Daniel Fernando Pereira Vasconcelos

**Affiliations:** 1 Universidade Federal do Delta do Parnaíba Laboratório de Análise e Processamento Histológico Parnaíba PI Brasil Universidade Federal do Delta do Parnaíba (UFDPar), Laboratório de Análise e Processamento Histológico (LAPHIS), Parnaíba, PI, Brasil.; 2 Universidade Federal de Pelotas Departamento de Morfologia Pelotas RS Brasil Universidade Federal de Pelotas (UFPel), Departamento de Morfologia, Pelotas, RS, Brasil.; 3 Faculdade de Medicina Afya Parnaíba PI Brasil Faculdade de Medicina (Afya), Parnaíba, PI, Brasil.; 4 Universidade Federal do Delta do Parnaíba Laboratório de Fisiologia-Farmacologia Experimental Parnaíba PI Brasil Universidade Federal do Delta do Parnaíba (UFDPar), Laboratório de Fisiologia-Farmacologia Experimental (LAFFEX), Parnaíba, PI, Brasil.

**Keywords:** Anti-Inflammatory agents, Antioxidants, Fatty Liver, Periodontal diseases

## Abstract

**Background:**

Periodontitis affects millions of people and is characterized by the accumulation of bacteria in the gingival sulcus with an immune-inflammatory response of the body causing effects. There is a notable relation between periodontitis and steatosis, in which caspase-8 may be a relevant player in the pathophysiology of these conditions. This study is the first to investigate the effect of treatment based on *Lactobacillus sp*. on steatosis and caspase-8 expression in a ligature-induced periodontitis model. This study aims to investigate whether treatment with *Lactobacillus sp.* reduces oral and hepatic changes caused by ligature-induced periodontitis.

**Methodology:**

Twenty-four Wistar rats were separated into groups: control, periodontitis, and periodontitis plus *Lactobacillus sp.* Administration of 1 mL of fermented milk containing 10^8^ CFU/mL of Lactobacillus sp. by gavage was performed daily for 20 days of ligature-induced periodontitis. After the treatment, we evaluated the gingival bleeding index (GBI), tooth mobility, probing pocket depth (PPD), alveolar bone loss, histomorphometry, and histopathological aspects of liver, as well as the levels of glutathione (GSH), malondialdehyde (MDA) and myeloperoxidase (MPO). We also evaluated caspase-8-positive cells and blood biomarkers.

**Results:**

*Lactobacillus sp.* reduced inflammatory clinical parameters, including GBI, PPD, and tooth mobility, as well as neutrophil infiltration in gingival tissue. Morphometric analysis showed significantly less alveolar bone loss. Analysis of hepatic tissue showed reduced neutrophilic infiltration and improved antioxidant activity. Treatment also decreased caspase-8 expression.

**Conclusions:**

*Lactobacillus sp.* significantly reduced the clinical parameters of periodontal lesion and steatosis score, improved hepatic oxidative status, and decreased caspase-8 expression in the liver tissue.

## Introduction

Periodontitis is characterized as a multifactorial chronic inflammatory disease that affects millions of people around the world. The disease is caused by the accumulation of bacteria in the gingival sulcus with an immune-inflammatory response of the organism.^1^ Some studies have shown that periodontitis is not only a local disease but is also associated with systemic diseases such as diabetes,^2^ respiratory diseases,^3^ liver disease,^4,5^ among others.

The literature describes the association between hepatic steatosis and periodontitis, showing that biochemical markers such as myeloperoxidase (MPO), malondialdehyde (MDA) and glutathione (GSH) have a close proximity^6,7^ in which the release of mediators of local inflammation and their access to the bloodstream have been described as one of the mechanisms for the development of liver injury, since such mediators increase oxidative stress and lipid peroxidation of the tissue.^5^

Also, bacteria present in the subgingival sulcus have a negative effect on the liver through inflammatory cytokines and bacterial toxins that gain access to the bloodstream.^8^ Previous data demonstrated the influence of *Porphyromonas gingivalis*, an important microorganism involved in the pathogenesis of the disease, on the progression of non-alcoholic steatohepatitis.^9^

The aspartate-specific cysteine protease (caspase) is a key player in the pathophysiology of liver alterations. Caspases are the main mediators of apoptosis, which are divided into initiator and effector caspases. Those that play a regulatory role are called initiators, such as caspase-8 and -9, whereas those that cleave several cellular substances are called effectors, such as caspases-3, -6, and -7.^10^

There are two signaling pathways for caspase activation: one involving receptor binding that leads to the recruitment of pro-caspase-8 into a death-inducing signaling complex, namely the extrinsic pathway, and the other that begins with the mitochondrial release of cytochrome C.^11^

The relation of caspase-8 and liver alterations may be defined by the role of tumor necrosis factor α (TNF-α) whose transduction is mediated by TNF-receptor 1 (TNFR1). The interaction between this mediator and its receptor culminates in the formation of a protein complex that activates the nuclear factor kB (NFkB) pathway and then the triggering of caspase-8.^12^

On the other hand, studies have shown that probiotics such as *Lactobacillus sp.* may be used as adjuvant treatment for gastrointestinal disease,^13^ cancer^14^and periodontitis.^15^ *Lactobacillus sp.* acts modulating host defense, stimulating endogenous antimicrobial production and competing with periodontopathogenic bacteria.^16^

Alterations in the composition and function of the gut microbiota have been associated with an increased risk of systemic diseases such as non-alcoholic fatty liver disease (NAFLD), indicating the existence of an oral-gut-liver axis.^17^ The molecular evidence of this axis is described by the role of microbial metabolites, such as short-chain fatty acids (SCFAs)^18^ and trimethylamine N-oxide (TMAO) ,^19^ and oxidative stress contributing to metabolic dysregulation.

It is well established that periodontitis may cause hepatic alterations; therefore, one can clearly see the importance of studies that specifically demonstrate the changes caused by periodontitis and possible treatments for this condition. Therefore, this study aimed to analyze the effects of the administration of *Lactobacillus sp.* in oral and hepatic tissues, evaluating caspase-8 expression, in a periodontitis model.

## Methodology

### Ethical issues and animals

All procedures and treatments were performed after approval by the Research Ethics Committee of the Animal Center Laboratory of the Federal University of Piaui (385/17). A total of 24 female *Wistar* rats (mean weight = 210g±1.6 g) were kept in a room with a temperature around 22±2°C with relative air humidity between 60 - 75%, light / dark cycles of 12 h. The animals had free access to water and feed and were kept for 7 days under these conditions to adapt to the environment.

### Experimental design

#### Bacteria isolation

*Lactobacillus sp.* was isolated from Chamyto^®^ (Nestlé Brazil Ltd.). The fermented milk was prepared following strict controlled conditions.^20^ The bacteria were isolated to avoid possible interference by other components of Chamyto^®^. The isolated bacterium was inoculated, after double activation (2% v/v inoculum, 37 °C, 24 h), in de Man, Rogosa and Sharpe broth (MRS, Laborclin, Paraná, Brazil), as a 2% v/v inoculum in sterile milk solution. This solution comprised skim milk powder 10% w/v (Molico^®^, Nestlé Brazil Ltd.) and sucrose 10% w/v P.A.-A.C.S. (Synth, Diadema, SP, Brazil) in distilled water. Fermentation lasted for 12 h at 37°C to reach the minimum count of 10^8^ colony forming units (CFU)/mL.

#### Sample Size Determination

To ensure robust detection of biologically significant group differences, the required sample size was estimated before study initiation using G*Power 3.1. The analysis was based on a one-way ANOVA design (fixed effects, global F-test), with the following specifications: significance level: α = 0.05 (two-sided), desired power: 95% (i.e., β = 0.05), and anticipated effect size: f = 0.6 classified as large per Cohen’s benchmarks. This effect size estimate was grounded in prior literature reporting outcomes in murine ligature-induced periodontitis models^21^ and corroborated by our internal pilot data, which replicated the same experimental conditions, surgical protocol, and primary endpoints. Under these assumptions, the calculation indicated that eight animals per experimental group would provide ≥95% power to identify the expected treatment effects. This number was therefore used as the target sample size.

The rats were separated into 3 groups (n = 8): Control group (no ligation), periodontitis (with ligature at the first molar) and periodontitis + *Lactobacillus sp.* (with ligature under the treatment with the solution of *Lactobacillus sp.*).

Periodontitis was induced after administration of general intramuscular anesthesia through an injection containing a solution of 16 mg/kg of 2% xylazine hydrochloride (Francotar-Virbac^®^, Roseira, SP, Brazil) and 40 mg/kg of ketamine (Rompum-Bayer®, São Paulo, SP, Brazil).

Subsequently, a nylon ligature was placed around the cervical region of the first lower molars and given a double reinforcement knot according to Vasconcelos, et al.^21^ (2017) for 20 days. In the treated group, *Lactobacillus sp.* administration was performed by gavage after the induction of periodontitis for 20 days. Daily 1 mL containing 10^8^ CFU/mL of the fermented milk was administered. After the treatment period, the animals were euthanized by intraperitoneal administration of an overdose of ketamine 300 mg / kg + xylazine 30 mg / kg. Blood was collected for proper biochemical procedures. Then, the total weights of animals and of organs were measured and described in absolute (g) and relative values (%, organ weight X 100 / body weight). The study was conducted in accordance with ARRIVE guidelines.

## Oral parameters

### Gingival Bleeding Index, Tooth Mobility and Probing Pocket Depth

The gingival bleeding index was measured for 10 seconds according to Liu, et al*.*^22^ in scores ranging from 0 to 5.

The tooth mobility was measured in the lower first molars at 0, physiological mobility; 1, slight mobility; 2, moderate mobility; and 3, pronounced mobility. Three points were measured and the mean was used^23^. Probing Pocket Depth was measured using a round-ended probe (tip with 0.2-mm radius) according to Liu, et al.^22^(2012).

## Myeloperoxidase (MPO) Activity

The analysis of MPO activity of the gingiva and liver was performed to indirectly determine the infiltration of neutrophils in the tissues following the methodology: concisely, 55 mg of tissue was homogenized at 55 mg/mL in potassium buffer containing 0.55% hexadecyltrimethylammonium bromide (HTAB). The homogenate was centrifuged at 4,000× g for 420 seconds at 5°C. The pellet was resuspended, and myeloperoxidase activity was assayed by measuring the change in absorbance at 450 nm, in ELISA reader, using o-dianisidine dihydrochloride and 1% hydrogen peroxide. Myeloperoxidase activity was described as units/mg of tissue. A unit of myeloperoxidase activity was defined as that converting 1 μmol of hydrogen peroxide to water in 60 seconds at 23°C^24^.

## Measurement of Alveolar Bone Loss

The measurements were made considering the delimitation of the cement-enamel junction. After dissection of the soft tissue, the mandibles were stained with 1% methylene blue. The alveolar bone height image for each hemimandible was captured using a stereomicroscope at a magnification of 30× according to Carvalho, et al*.*^25^(2017).

## Malondialdehyde (MDA) and Glutathione (GSH) levels of the liver

MDA was used as an indicator of lipid peroxidation. The MDA concentration for liver tissue was measured using the method described previously. The solution was homogenized and 250 μl of 10% tissue was prepared in 1.16% KCl. Then, 3 mL of 0.55% thiobarbituric acid solution were added to 500 μl of the homogenate in a tube. This mixture was placed in a water bath for 46 min at 100°C, 4 milliliters of n-butanol was added, and the mixture was centrifuged. Absorbance was measured at 520 nm (A1) and 532 nm (A2), in which the amount of malondialdehyde was calculated as (A2-A1), and expressed as nmol MDA per g of liver tissue.^26^

GSH levels were determined according to the method described by Sedlak and Lindsay^27^(1968). The liver tissue was homogenized in 250 μl of 0.02M ethylenediaminetetraacetic acid (EDTA), to obtain a 5% tissue solution. Then, 315 μL of distilled water and 88 μL of 55% trichloroacetic acid (TCA) were added; the samples were centrifuged at 3,000 rpm for 0.4 h at 4°C. Part of the supernatant (440 μL) was added to 880 μL of 0.4M Tris buffer at pH 8.8 with 25 μL of 0.01M 5,5′-dithiobis(2-nitrobenzoic acid) (DTNB). Subsequently, the absorbance of each sample was evaluated at 412 nm, and the GSH data were obtained and expressed per mg of tissue.^27^

## Histopathological evaluation of the liver

Samples were collected and fixed in 10% buffered formaldehyde. After the inclusion process and cuts of 6-μm thickness, the samples were stained with hematoxylin and eosin for analysis under light microscopy. Histological evaluation of hepatic tissue inflammation, steatosis and necrosis was graded and analyzed. Hepatic samples were preserved in formaldehyde following euthanasia, and histologic processing was performed for the left lobe. Five-micrometer-thick sections were prepared and stained with eosin and hematoxylin. Each liver was represented by 15 sections, examined at 600× magnification on a light microscope. Histological evaluation of hepatic tissue followed the parameters of (a) hepatic steatosis, (b) inflammatory changes, and (c) necrosis. The steatosis score was determined and classified according to the percentage of hepatocytes containing steatosis, using a five-grade scale: 0 = absent or present in 0–4% of fields; 1 = present in 5–25% of cells; 2 = present in 26–50% of cells; 3 = present in 51–75% of cells; 4 = present in >75% of cells.^21^

## Blood Biomarkers

We performed alkaline phosphatase, urea, and creatinine measurement in blood following the procedure indicated by the commercial Labtest kits.

## Caspase Assay

A monoclonal mouse anti-caspase-8 antibody was used for evaluation of the caspase-8 expression (Novocastra™ Peroxidase Detection System, RE7101). Based on the antibody specifications, the samples were incubated with the primary antibody at a concentration of 1:30 for 1 hour, at room temperature, in a humid chamber. The negative control consisted of coverslips incubated with TBS+BSA 2%. Biotinylated secondary anti-IgM and anti-IgG antibody (Novocastra™ Peroxidase Detection System, RE7101), in Tris-buffered saline, with protein stabilizer and 0.35% ProClin™ 950, was used directly on the coverslips, and incubated for 30 minutes at room temperature, in a humid chamber. All cells that showed a brownish color with clear and homogeneous cytoplasmic contours were considered positive for caspase-8 protein as described by Silva, et al.^28^(2026).

## Statistical Analyses

The data from the assays are expressed as mean ± S.E.M. and/or median. The Shapiro-Wilk test was performed to verify the distribution of the data. Differences among the groups were investigated by means of the Kruskal-Wallis test, for values with non-normal distribution, and the ANOVA test for normally distributed values; in both cases the value p<0.05 was considered significant. In figures, * indicates a statistically significant difference. Statistical tests were performed on a specific statistical software (GraphPad Prism Software Version 5.0), and the p-value was considered significant when <0.05.

## Results

### Clinical Outcomes

**Figure 1:**
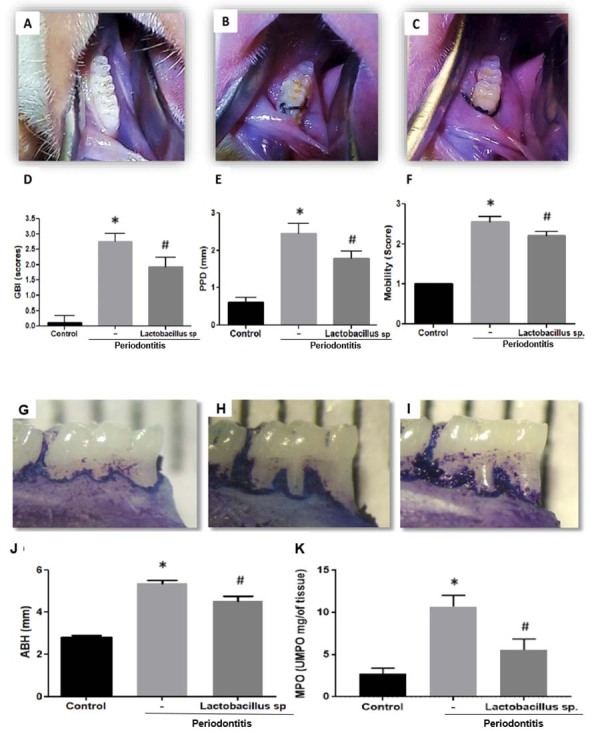
Shows A as the lower molar of the control group presents healthy periodontium. Image B shows the group of periodontitis of the lower molars with the presence of ligation associated with edema and bacterial plaque around the lower first molar. Image C shows the lower molar of the *Lactobacillus sp*. with reduced plaque presence, bleeding into the gingival sulcus, and color change. Images D, E and F show, respectively, the values of Gingival Bleeding Index, Probing Pocket Depth and TM, where both experimental groups, periodontitis and *Lactobacillus sp*., presented a statistically significant difference in relation to the control group. The images G, H and I represent the clinical difference of the alveolar bone. G Control group, without change. H Periodontitis group presenting high alveolar bone loss and I Lactobacillus sp. demonstrating moderate bone loss. J shows the statistical difference found in the alveolar bone height measurements and image K, which shows the gingival MPO dosing result, where significant statistical differences can be observed when compared among the three groups, * p<0.05 vs control group, and # p<0.05 vs periodontitis group.

The periodontitis group presented in the region where the disease was clinically manifested intense edema, color change, a great accumulation of bacterial plaque and intense gingival bleeding. The *Lactobacillus sp.* group showed a significant clinical reduction in the amount of bacterial plaque and moderate gingival bleeding as shown in (Figure 1A, 1B, 1C).

### Oral parameters

#### Gingival Bleeding Index, Probing Pocket Depth, Tooth Mobility and Myeloperoxidase

GBI and PPD were significantly lower in the *Lactobacillus sp.* group when compared to the periodontitis group, but there was no statistically significant difference in TM. (GBI: Control, 0.11±0.07; Periodontitis 2.75±0.08; *Lactobacillus sp.*, 1.92±0.11; PPD: Control, 0.61±0.13; Periodontitis 2.15±0.37; *Lactobacillus sp.*, 1.79±0.18; TM: Control, 1.0±0.0; Periodontitis 2.45±0.2; *Lactobacillus sp.*, 2.28±0.26; p<0.05) (Figure 1D, 1E, 1F).

Gingival MPO was significantly lower in the *Lactobacillus sp.* group (Control: 2.7 ± 0.6, periodontitis: 10.7±1.3, *Lactobacillus sp.*: 5.5±1.2, p <0.05) (Figure 1K).

## Measurement of Alveolar Bone Height (ABH)

Figures 1 G, H and I show the difference between the alveolar bone in control, periodontitis and *Lactobacillus* sp groups. There were statistically significant differences (control, 2.81±0.71 mm; periodontitis, 5.35±0.14 mm, *Lactobacillus sp.* 4.529±0.23 mm; p<0.05) between the control and periodontitis groups, with higher ABH values for the periodontitis group, which were reduced in the group treated with *Lactobacillus sp.* (Figure 1J).

## Myeloperoxidase, Glutathione and Malondialdehyde

**Figure 3:**
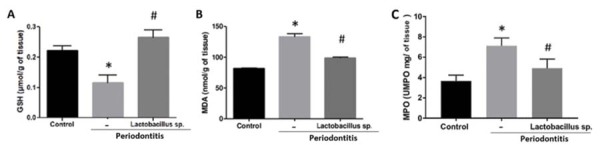
The A and B images represent the means and standard deviation of the GSH and MDA dosages of hepatic tissue, respectively. C shows values of the MPO levels of the liver, showing statistically significant differences among the groups evaluated, * p<0.05 vs control group, and # p<0.05 vs periodontitis group.

The MPO of the liver showed a statistically significant difference between the groups (control: 3.66±0.33; periodontitis: 7.13±0.28; *Lactobacillus sp.*: 4.91±0.40, p<0.05) (Figure 3C). The GSH of the liver was statistically significant (p<0.05) with higher levels in the *Lactobacillus sp.* group (0.26±0.01) when compared to the group with periodontitis (0.11±0.01). Control values were (0.22±0.00) (Figure 3A). The hepatic MDA dosage was statistically significant (p<0.05) with reduced levels in *Lactobacillus sp.* (98.59±1.77) when compared to the group with periodontitis (133.00±5.11). Control values were (81.52±1.02) (Figure 3B).

## Histopathological evaluation of the liver

**Figure 2:**
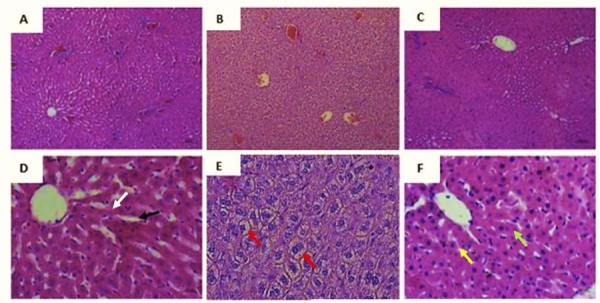
A and D represent the hepatic tissue of the control group without histological changes, hepatocytes in normal conformation. B and E represent the hepatic tissue of the periodontitis group demonstrating several hepatocytes with loss of conformation, presenting steatosis. C and F show the hepatic tissue of the Lactobacillus sp. group which exhibit less altered hepatocyte cord conformation than the periodontitis group, and a reduction in steatosis.

The liver tissue that received the administration of *Lactobacillus sp.* showed a significant reduction in the prevalence of hepatic steatosis (Table 1) when compared to the periodontitis group. The hepatocytes of *Lactobacillus sp.* group had a normal structure in the organization and between the cords and the sinusoids did not present blood congestion, as it was observed in the periodontitis group. The liver tissue of the control group did not show significant histological changes (Figures 2A-F). Inflammation and necrosis also showed significant changes (Table 1).

**Table 1:** Histopathological assessment of the liver and positive cells for caspase-8

Parameters assessed	Groups		
	Control	Periodontitis	*Lactobacillus* sp
Steatosis score	0 (0 - 1)	2 (1 - 3)*	1 (0 - 1)^†^
Inflammation score	0 (0 - 1)	1 (0 - 1)	0 (0 - 1)
Necrosis score	0 (0 - 1)	1 (0 - 1)	0 (0 - 1)
Positive cells for caspase-8	1.2 ± 1.0	5.6 ± 1.4*	2.9 ± 1.2^†^

* Indicates statistical significance between periodontitis group versus control group (P < 0.05) and † also indicates the statistical difference between periodontitis group versus *Lactobacillus sp.* (P < 0.05). Statistical analysis: Kruskal Wallis test, followed by Dunn’s test, was used scores for steatosis, inflammation and necrosis. ANOVA followed by Student-Newman-Keuls test for caspase-8.

## Weight parameters and blood levels

There was a statistically significant difference (p <0.05) in the *Lactobacillus sp.* group when compared to the periodontitis group in the following blood markers: alkaline phosphatase and albumin. There was no statistically significant difference in the dosages of urea and creatinine. All of these markers are described in Table 2.

**Table 2:** Weight parameters and blood biomarkers

Parameters Assessed	Groups		
	Control	Periodontitis	*Lactobacillus* sp
**Weight**			
Body	219 ± 4.3	219 ± 4.2	227 ± 8.1
**Liver**			
Absolut (g)	7.0 ± 0.2	8.0 ± 0.2	8.0 ± 0.1
Relative (%)	3.27 ± 0.04	3.84 ± 0.07	3.5 ± 0.1
**Blood biomarkers**			
Alkaline phosphatase	58.76 ± 4.6	84.29 ± 14*	45.5 ± 3.8^†^
Albumin	3.4 ± 0.08	2.2 ± 0.2*	3.0 ± 0.2^†^
Creatinine	0.32 ± 0.03	0.25 ± 0.05	0.25 ± 0.02
Urea	52.13 ± 1.9	40.5 ± 1.3	47.7 ± 2.8

* indicates statistical significance between periodontitis group versus control group (P < 0.05) and † also indicates the statistical difference between periodontitis group versus *Lactobacillus sp*. (P < 0.05). Statistical analysis: ANOVA followed by Student-Newman-Keuls test.

## Caspase Assay

**Figure 4:**
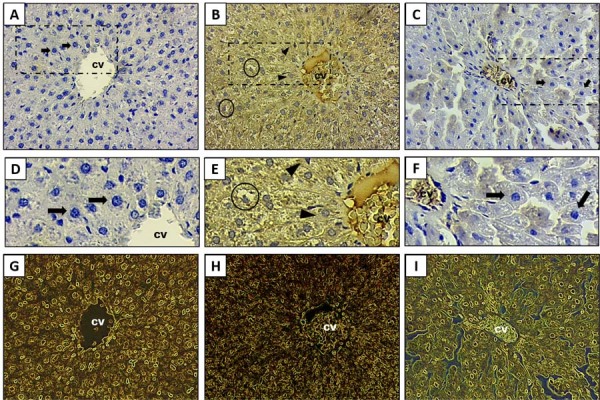
The image shows the immunohistochemistry of the liver of caspase-8 positive cells. A shows the normal liver of the control group, with the central vein (CV) and normal hepatocytes (arrows). B shows the liver of the periodontitis group with marking of caspase-8 positive cells (arrow-head) and showing microvesicular steatosis (circles). C shows the group treated with *Lactobacillus sp.* with a marked decrease in caspase-8 positive cells and microvesicular steatosis. D, E and F show the same images as A, B and C respectively at a higher magnification. G, H and I show the same image as A, B, and C with a filter to highlight the differences.

In Table 1 and Figure 4, the result of the analysis of the caspase-8 positive cells in the liver (cells / 45 × 10^3^ µm^2^) is shown, in which we can observe that the treatment with *Lactobacillus sp.* decreases the concentration of caspase-8 levels in the tissue when compared to the periodontitis group (control, 1.2 ± 1.0 cells/µm^2^; periodontitis 5.6 ± 1.4 cells/µm^2^; *Lactobacillus sp.*, 2.9 ± 1.2 cells/µm^2^; p <0.05) .

## Discussion

To the best of our knowledge this is the first study to investigate the association among periodontitis, hepatic alterations, and the administration of *Lactobacillus sp.* as a probiotic*.* The results showed that *Lactobacillus sp.* treatment was able to decrease inflammatory clinical parameters such as GBI and PPD, and neutrophil infiltrate as shown by the reduced levels of MPO in the gingival tissue. Morphometric analysis demonstrated that there was a statistically significant reduction in alveolar bone height.

Treatment with *Lactobacillus sp.* had a global anti-inflammatory effect on all clinical indicators, which were reduced in this study (GBI, PPD and gingival MPO). These data are in accordance with Alaei, et al*.*^29^(2023), who demonstrated that *Lactobacillus sp.* can exert an anti-inflammatory effect *in vivo* by modulating immune responses and possibly acting via inhibition of pro-inflammatory cytokines.

MPO levels of the gingival and hepatic tissues were reduced. Hence, it is considered that there was a reduction of the neutrophil infiltrate in these tissues, as well as a reduction of cell apoptosis via caspase-8.

Our results agree with the study of Soares, et al.^30^ (2019) who used *Lactobacillus sp.* orally in patients for the treatment of periodontitis. There was a reduction in probing pocket depth and bleeding on probing (p<0.01) when compared to a placebo group.

In addition, analysis of liver tissue showed that there was also a reduction in steatosis scores in the *Lactobacillus sp.* group, presenting an antioxidant activity that can be confirmed by means of MDA and GSH dosages of hepatic tissue, as well as a minor neutrophilic infiltration.

Previous studies already demonstrated that periodontitis may have direct and indirect relationships in the development of hepatic steatosis.^4,5,21^ In our study it was not different, as the periodontitis group presented high steatosis scores when compared to the control group, while the group treated with *Lactobacillus sp.* showed a reduction in these scores.

It is interesting to note the slight increase in liver weight in periodontitis and *Lactobacillus sp.* groups (Table 2). This may be explained by the influence of periodontitis on hepatic tissue, possibly related to the high degree of steatosis. Periodontitis triggers chronic inflammation that can lead to systemic alterations, including decreased albumin levels, corroborating previous results.^4^

On the other hand, there was a significant decrease in albumin levels among the groups, with the periodontitis group showing the lowest values. This finding is in line with other data in the literature showing that rats with apical periodontitis presented low levels of serum albumin.^31^ Albumin has the capacity to bind reactive oxygen species,^32^ and serum albumin levels have been suggested as an indirect target for oxidative stress and periodontal tissue damage, especially in its form as ischemic modified albumin.^33^

Our data showed that *Lactobacillus sp.* has antioxidant potential, since there was a significant reduction of MDA levels and increase in GSH levels when compared to periodontitis and *Lactobacillus sp.* in the dosages performed in hepatic tissue, as well as a minor neutrophilic infiltration.

These data are in agreement with Olotu, et al.^34^(2024), demonstrating that *Lactobacillus sp.* has the potential to significantly reduce hepatic injury, inflammation, and proinflammatory cytokines, as well as increase antioxidant potential, resulting in non-activation of the caspase-8-mediated apoptosis pathway. Some data have already found that *Lactobacillus sp.* can be potentially toxic molecules depending on the concentration or tissue clearance, as *Lactobacillus sp.* could modulate the immune response.^35^

The treatment with *Lactobacillus sp. also* showed that there was a reduction in the expression of caspase-8 in liver cells, which means that there is a decrease in death signaling, since caspase-8 is contained in the subfamily of apoptosis activators.

The formation of death-inducing signaling complex (DISC) can initiate apoptosis through an extrinsic pathway induced by extracellular stimuli. Activated caspase-8 leads to a cascade of caspase-3 activation and initiates the mechanisms of apoptosis; in some cells, caspase-8 can cleave caspase-3 directly, not requiring the action of mitochondria, but in cases where caspase-8 needs to cleave Bid, a member of the Bcl-2 family, into tBid, which will subsequently induce mitochondrial outer membrane permeabilization.^36^

Recent studies show the relationship of periodontitis with the activation of caspases in its pathophysiology, showing that caspases are directly related to disease activation, since they lead to cellular apoptosis culminating in the destruction of periodontal tissues.^37-39^

Due to changes in liver cell structures, the expression of caspase-8 in hepatocytes was evaluated, where a decrease in its signaling was observed in the group treated with *Lactobacillus sp.*, which means that the treatment can decrease a death-signaling protein, possibly due to the increase in anti-apoptotic proteins Bcl-2, which binds to its specific receptor, preventing cytochrome c from binding to the activating factor.

Injury and cell death can be triggered when lipids accumulate inside non-adipose cells, entering a deleterious non-oxidative pathway; programmed death in hepatocytes is a common alteration in several pathologies that affect the liver,^40^ as in our experiment, where the induction of periodontitis leads to the appearance of microvesicular steatosis. Hepatocyte apoptosis occurs primarily through three convergent pathways: the mitochondrial pathway, the endoplasmic reticulum pathway, and the death receptor pathway. In our study, we demonstrated that reduced caspase-8 expression significantly attenuates this extrinsic apoptosis pathway.

An overexpression of Fas leads to a death domain, which forms a death-inducing signaling system, thereby activating procaspase-8, which regulates caspase-8, which in turn activates caspase-3, which acts on structural elements of the cell, causing its destruction and death.^41^ In our study, the use of *Lactobacillus sp.* prevented the activation of procaspase-8, preventing the cascade caused by caspase-8.

Thus, our study showed a decrease in caspase-8 expression in liver cells, after damage caused by periodontitis, where the administration of *Lactobacillus sp.* was effective as a liver protector, findings similar to those of Zhang, et al.^42^(2020), who evaluated caspase-8 expression in non-alcoholic fatty disease, showing that *Lactobacillus sp.* has an inhibitory action on the extrinsic pathway of apoptosis, thus preventing changes in liver tissue.

In addition, Sun, et al*.*^43^(2018), showed that *Lactobacillus sp.* reduces the inflammatory response and oxidative stress, aiding in the biosynthesis of GSH and reducing the levels of MPO, MDA and reducing the expression of pro-inflammatory genes. This result agrees with the reduction of liver MPO in *Lactobacillus sp*. However, more efforts are needed to understand the relationship of the mechanisms that involve the systemic effect caused by periodontitis. Although there are many studies, the exact relationship between the intestinal microbiota and the systemic effects in humans, mainly related to metabolic activity, is still unclear.

## Conclusions

Our data showed that prophylactic administration of *Lactobacillus sp*. significantly attenuated the progression of periodontal disease in rats with ligature-induced periodontitis. Specifically, there was a decrease in gingival inflammation, evidenced by a lower GBI in the treated group, reduced probing depth, and lower MPO activity in gingival homogenates. Hepatic steatosis was significantly attenuated, accompanied by restoration of redox homeostasis, including elevated GSH levels, reduced MDA concentration, and decreased caspase-8 expression. These results indicate that *Lactobacillus sp.* exerts simultaneous periodontal anti-inflammatory and systemic hepatoprotective effects in this model, supporting its investigation as an adjuvant strategy targeting the microbiota in comorbidities associated with periodontitis.
